# Smartphone Application for the Analysis of Prosodic Features in Running Speech with a Focus on Bipolar Disorders: System Performance Evaluation and Case Study

**DOI:** 10.3390/s151128070

**Published:** 2015-11-06

**Authors:** Andrea Guidi, Sergio Salvi, Manuel Ottaviano, Claudio Gentili, Gilles Bertschy, Danilo de Rossi, Enzo Pasquale Scilingo, Nicola Vanello

**Affiliations:** 1 Dipartimento di Ingegneria dell’Informazione, University of Pisa, Via G. Caruso 16, Pisa 56122, Italy; E-Mails: d.derossi@centropiaggio.unipi.it (D.R.); e.scilingo@centropiaggio.unipi.it (E.P.S.); nicola.vanello@unipi.it (N.V.); 2 Research Center “E. Piaggio”, University of Pisa, Largo L. Lazzarino 1, Pisa 56122, Italy; 3 Life Supporting Technologies, Universidad Politécnica de Madrid , Avd. Complutense 30, Madrid 28040, Spain; E-Mails: ssalvi@lst.tfo.upm.es (S.S.); mottaviano@lst.tfo.upm.es (M.O.); 4 Department of Surgical, Medical, Molecular Pathology and Critical Care, University of Pisa, Via Savi 10, Pisa 56126, Italy; E-Mail: c.gentili@unipd.it; 5 Department of General Psychology, University of Padua, Via Venezia 8, Padua 35131, Italy; 6 Department of Psychiatry and Mental Health, Strasbourg University Hospital, INSERM U1114, Translational Medicine Federation, University of Strasbourg, Strasbourg 67000, France; E-Mail: gilles.bertschy@chru-strasbourg.fr

**Keywords:** smartphone application, fundamental frequency, voice segmentation, pitch strength, voice monitoring system, bipolar disorders

## Abstract

Bipolar disorder is one of the most common mood disorders characterized by large and invalidating mood swings. Several projects focus on the development of decision support systems that monitor and advise patients, as well as clinicians. Voice monitoring and speech signal analysis can be exploited to reach this goal. In this study, an Android application was designed for analyzing running speech using a smartphone device. The application can record audio samples and estimate speech fundamental frequency, $F_{0}$, and its changes. $F_{0}$-related features are estimated locally on the smartphone, with some advantages with respect to remote processing approaches in terms of privacy protection and reduced upload costs. The raw features can be sent to a central server and further processed. The quality of the audio recordings, algorithm reliability and performance of the overall system were evaluated in terms of voiced segment detection and features estimation. The results demonstrate that mean $F_{0}$ from each voiced segment can be reliably estimated, thus describing prosodic features across the speech sample. Instead, features related to $F_{0}$ variability within each voiced segment performed poorly. A case study performed on a bipolar patient is presented.

## 1. Introduction

Mood disorders, especially bipolar disorders, have a great impact on people’s lives, and currently, large efforts are being made both to determine their causes and to improve therapy [1,2,3]. Bipolar disorders are defined as the periodical oscillations of pathological moods, eventually alternating between euthymic or normal states [4,5]. Depressive mood states can include feelings of sadness, low energy, insomnia, anhedonia, weight loss, psychomotor retardation, thoughts of fault, inadequacy and ideas of death and suicide. Manic periods (or hypomanic when the intensity of the symptoms are less severe) instead are marked by inflated self-esteem or grandiosity, increased energy, decreased need for sleep, increased talkativity, subjective experience that thoughts are racing or flying away, psychomotor agitation and increased risk-taking behaviors. Finally, a mixed state is defined when a patient experiences both manic and depressive symptoms at the same time. The possibility of monitoring a subject’s status and predicting depressive symptoms would allow one to modulate interventions, thereby minimizing the risks for the patients [6,7]. At the European level, several projects have been funded to tackle this issue, offering different approaches, such as monitoring physiological parameters and using behavioral questionnaires [3,8,9,10]. Speech signal analysis can provide further information about the mood state of a patient. In fact, speech production is a complex phenomenon that is influenced by the autonomic and somatic nervous systems, through the modulation of breathing activity, vocal muscles tension, salivation and mucus secretion [11,12,13]. This observation led research towards the exploration of correlations between mood state and voice or speech signals in mood disorders. Voice-related features were found to have a predictive value for developing depressive symptoms in adolescents [14]. A relationship between speech-related features describing prosodic changes and depression severity was observed [15]. Glottal, prosodic and vocal tract features could discriminate depressed subjects with respect to controls [16]. Glottal-flow spectrum and vocal jitter were found to discriminate near-term risk suicidal subjects [17]. Moreover, both an increase and a decrease of the average speech fundamental frequency, $F_{0}$, as well as its variability were observed. This ambiguous phenomenon was hypothesized to be related to different forms of depression, *i.e.*, agitated or retarded. The same phenomenon was observed in [18], where opposite trends were observed in average $F_{0}$, $F_{0}$ standard deviation and jitter, estimated in voiced segments. These observations may indicate the need for a personalized approach for the analysis of speech features in mood disorders to improve the performances of a decision support system that informs the physician about a patient’s mood change. Such a system could be used by patients while at home. Regarding voice recording devices, different solutions have been proposed. In [19], a portable system for the acquisition and processing of speech samples was introduced. The system consists of a mini computer equipped with a microphone and keyboard, and it was developed with the aim of detecting voice quality in people suffering from Parkinson’s disease and to give them consistent feedback about speech therapy progress. In [20], the performance of an accelerometer and a contact microphone were evaluated for the design of a voice analyzer. The accelerometer was found to be more robust with respect to environmental noise than the contact microphone, although a smaller bandwidth was present. A smartphone device and an accelerometer were the key elements of the device shown in [21]. This system was shown to be robust against noise and could estimate sound pressure level, as well as the glottal airflow-based measure and fundamental frequency. A wearable voice accumulator based on a pocket PC and an accelerometer was described in [22]. In this case, the authors confirmed that the robustness against environmental noise was high, but the system was sensitive to the subject’s movements. In [23], a mobile phone was used to collect speech data both using structured and unstructured tasks, with the aim of classifying the mood state in bipolar disorders. Speech data were collected with a sampling frequency of 8 kHz and sent to a central server for the feature extraction process. The reliability of the estimated features was not indicated, and it is not clear whether the overall process is automatic. On the other hand, a complex feature extraction and classification procedure is discussed, and preliminary results indicate good classification of hypomania, while lower performance was obtained with depression.

In this study, we explore the possibility of using a smartphone to collect and process speech data for the estimation of features related to the speech $F_{0}$ and its variability measured over voiced segments. The features are estimated locally, on the smartphone, exploiting an algorithm based on a spectral matching approach [24], and sent to a server, thus guaranteeing subjects’ privacy. The performance of the proposed approach is compared to that achievable with a high quality recording system. A case study involving a bipolar subject is presented.

## 2. Experimental Section

The smartphone was used to collect and process speech data via an Android application. In this section, the developed application along with the procedures for testing its performance are described. The tests were designed to evaluate the audio quality and the performance of the algorithm running on the smartphone. A longitudinal study on a bipolar patient is introduced. Specifically, the procedures for the clinical assessment, the speech task and a correlational analysis among speech features and observed mood changes are described.

### 2.1. Application

**Figure 1:**
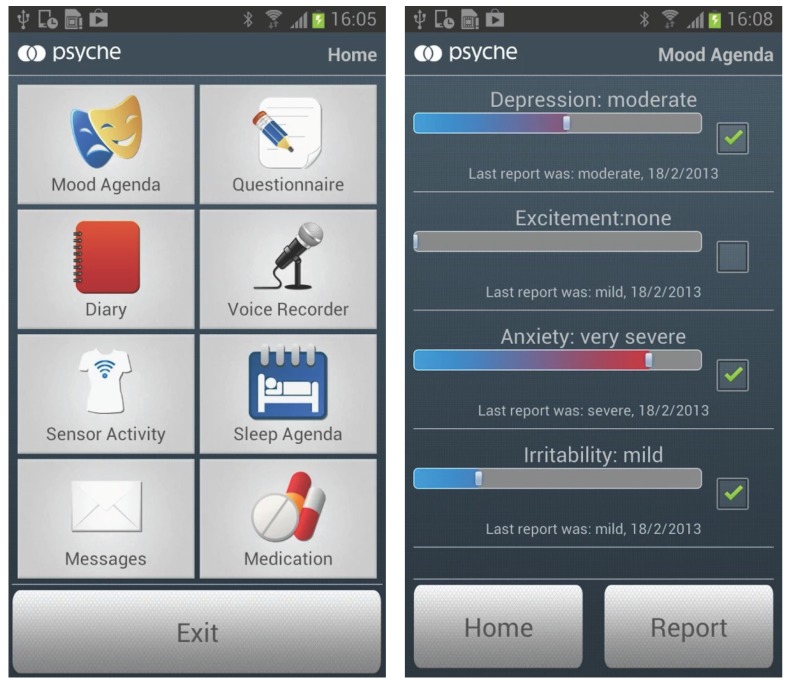
Screenshots of the developed application: main menu (left), and an example of the mood agenda (right).

The Android application is integrated in a software tool of the PSYCHE Platform System [8]. This system is developed to monitor the mood states of subjects suffering from bipolar disorders. The system allows for compiling a mood agenda, answering daily self-administered questionnaires, storing data coming from a physiological signal monitoring system and acquiring and analyzing speech data [25]. In Figure 1, two screenshots of the application main menu and an example of a mood agenda are shown.

The application is also able to record speech data using two structured tasks. In the first task, the subject is asked to read text, while in the second, to comment on some pictures. Both text and images were selected by the clinicians participating in the study. The text is the Declaration of Human Rights that is supposed not to elicit any strong emotional reaction. The pictures depict ambiguous situations, thus pushing the subjects to interpret them. Subjects can select the task and decide when to start its execution. The task was set to be equal to 30 s long. At the end of the acquisition, speech data are stored in the memory of the smartphone, and they are automatically processed in the background to estimate the vocal fundamental frequency $F_{0}$ over time. This step is accomplished using a variation of the sawtooth waveform inspired pitch estimator algorithm (SWIPE’) [24], which estimates $F_{0}$ at given time instants using a spectral matching approach. The SWIPE’ algorithm was recently shown to have very good performance as compared to established algorithms [26]. This algorithm estimates $F_{0}$ as the fundamental frequency of a sawtooth waveform whose spectrum best approximates the speech signal spectrum. Moreover, this algorithm estimates the pitch strength, that is the measure of similarity between the speech spectrum and the sawtooth model [27]. This parameter plays an important role for the detection of voiced segments where speech features can be estimated. The application then sends the extracted features, as a text file, to a central server, where they are further processed. The remote processing phase is focused on both the detection of voiced segments and the extraction of the final features. Voiced segments are defined as those having a pitch strength higher than a given threshold. An investigation was performed to detect a proper threshold value, according to given criteria. Specifically, we chose a threshold to reduce the probability of labeling an unvoiced segment as voiced, *i.e.*, increasing voiced segment detection specificity, at the expense of a higher probability of missing voiced segments, *i.e.*, reducing sensitivity. The investigated final features are the mean $F_{0}$, jitter and $F_{0}$ standard deviation ($stdF_{0}$) estimated in each voiced segment. Camacho’s SWIPE’ algorithm was implemented in Android by porting the C code developed by Kyle Gorman and reported in [28]. Such porting was made possible by the Java Native Interface [29]. For this purpose, two libraries were linked to the application for fast Fourier transform [30] and managing sound files in wav format [31].

### 2.2. Testing of the System

The performance of the proposed system were evaluated considering both the quality of the speech acquisitions and the reliability of the algorithm implemented on the Android platform. Moreover, the reproducibility of the results was evaluated adopting two different positions of the device with respect to the user. To achieve these goals, different tests were designed using as ground truth values the results obtained with a high quality audio system and a validated algorithm for $F_{0}$ estimation. Two healthy subjects, one female (25 years old) and one male (30 years old), were enrolled for system testing. Each subject was recorded for about 30 s. The high quality acquisition system was formed by an AKG P220 Condenser Microphone and an M-Audio Fast-Track acquisition board. A sampling frequency of 44.1 kHz and a resolution of 32 bits were adopted. The smartphones used were two Samsung I9300 Galaxy S III. The audio data were acquired by the smartphones using a sampling frequency of 48 kHz and 32-bit resolution without any compression. We will refer to the high quality acquisitions as HQmic. The high quality and the smartphone recordings were obtained simultaneously. Two different positions of the smartphone with respect to the user were tested. Both positions allowed the user to read text or comment on the images displayed by the smartphone application while assuring a good recording quality. Specifically, two smartphones were used in each acquisition: one was kept on a table in front of the subject (${\text{SPmic}}_{\text{table}}$), while another one was held by the subject (${\text{SPmic}}_{\text{hand}}$). Both the microphone and smartphone held by the subject were positioned in front of the subject, at 30 cm from her/his mouth, symmetrically with respect to the face. The smartphone on the table was kept in front of the subject at a 40-cm distance from the subject’s mouth. The outcomes of an algorithm implemented in MATLAB [32] and running on a personal computer were used as the reference, *i.e.*, ground truth measures. This algorithm will be indicated as GTal (Ground Truth algorithm) and is formed by a two-stage procedure. In the first step, voiced segments are detected using both signal intensity and zero crossing rate as in [18]. This approach was also tested in [32] and compared to the segmentation that can be obtained using an electroglottographic signal. In the second step, the estimates of $F_{0}$ are obtained using an algorithm based on SWIPE’, whose good performance was shown in [33]. An additional smartphone (LG Nexus 4 E960) was used to investigate a possible change in the results when using another device, which may have a different audio quality. For this purpose, dedicated simultaneous acquisitions were performed by using the two different smartphones. Both smartphones were held close together by the subject.

#### 2.2.1. Audio Quality Testing

Dedicated tests were designed to evaluate the quality of the smartphone audio acquisitions with respect to those obtained with the high quality recording device. These results will allow one to verify whether the audio recording quality could affect final feature reliability. For this reason, the high quality algorithm, GTal, was applied to the audio file obtained with the high quality microphone, HQmic, and those obtained with the smartphones, ${\text{SPmic}}_{*}$. All audio files were uploaded and processed on the same personal computer.

#### 2.2.2. Algorithm Reliability

The aim was to evaluate the performance of the algorithm implemented on the Android platform and running on the smartphone. We will refer to this algorithm as ANDal (Android algorithm).

To achieve this goal, the results of the ANDal algorithm were compared to those achieved with the GTal. Both algorithms were applied to the recordings obtained using the high quality systems. This comparison was made possible by uploading high quality data to an Eclipse Integrated Development Environment (IDE) virtual machine running the ANDal algorithm. In fact, the results obtained with the virtual machine are exactly the same as those obtained using the smartphone.

#### 2.2.3. Overall System Testing

To test the performance of the overall system, the outcomes of the smartphone application, *i.e.*, ${\text{SPmic}}_{*}$ + ANDal, were compared to those obtained with the best reachable setup in a structured environment, *i.e.*, HQmic + GTal. A preliminary evaluation of the device-dependent variability of the overall system performance was also introduced. Specifically, the results of the applications running on the two different models of smartphone devices were compared using concurrent acquisitions.

#### 2.2.4. Statistical Analysis

For each of the above-described comparisons, both voiced segment detection performance and estimated feature reliability were evaluated. The voiced segmentation approach performance was assessed looking at segment length and defining sensitivity and specificity parameters using the segments obtained from high quality data as a reference. The evaluation of the estimated features was done both by looking at the feature distributions across segments and by correlating the measures estimated from voiced segments in the two conditions. Regarding the former evaluation, given the non-Gaussian distribution of the features, a two-sample Kolmogorov–Smirnov test was adopted, and a significance level equal to 0.05 was adopted. The correlational analysis was performed taking into account only overlapping portions of the segments. The threshold for the significance of the correlation coefficient was set to p=0.05.

### 2.3. Patient Data

A pilot study was performed on a patient suffering from bipolar disease to show the potentialities of such an application. The patient, a 36-year-old female, was able to lead an independent and active life in absence of any suicidal tendencies, or delusions, or hallucinations and did not suffer from any substance use disorders. The patient was recruited at the Department of Psychiatry and Mental Health of the Strasbourg University Hospital under the PSYCHE protocol and had a diagnosis of Type II bipolar disorder according the Diagnostic and Statistical Manual of Mental Disorders, 4th edition, text revision (DSM-IV-TR) criteria. Type II bipolar disorder is a condition in which there are depressive and hypomanic (but not manic) episodes. The patient signed an informed consent to participate in the study approved by the Ethical Committee of the Strasbourg University Hospital. The patient was taking a mood stabilizer (lithium), antipsychotics (aripiprazole, cyamemazine) and an anxiolytic (alprazolam). The system was given to the patient for 14 weeks, and the picture commenting task was performed 15 times while at home. The patient’s mood state was assessed by the clinician during the day before each voice recording session. The assessment was done by a boarded physician or clinical psychologist using the Quick Depression Inventory (QID) for Depression and the Young Mania Rating Scale (YMRS). A score equal to or higher than 8 on the QID was considered an index of depressive state, and a score equal to or higher than 6 on the YMRS was considered an index of hypomanic state. If both of the scores exceeded the cut-offs, the patient was considered in a mixed state. Finally, if both of the scores were under the cut-off, the patient’s state would have been labeled as euthymic (*i.e.*, in clinical remission). The system was able to return as an output some statistics summarizing the distribution of $F_{0}$-based features across voiced segments detected in each acquisition. Specifically, the median, the median absolute deviation (MAD) and the skewness were estimated. The correlational study about the mood state and the above-mentioned summary statistics was performed by means of the Spearman approach.

## 3. Results and Discussion

### 3.1. Audio Quality Testing

The analyses of the data acquired from the two subjects (Subj. 1 and Subj. 2) revealed no statistically-significant differences in any of the three investigated features, *i.e.*, $meanF_{0}$, $stdF_{0}$ and Jitter. Noticeably, no statistically-significant differences were detected by comparing the results obtained with the smartphones in different positions, *i.e.*, ${\text{SPmic}}_{\text{table}}$ and ${\text{SPmic}}_{\text{hand}}$.

In Table 1, the median and median absolute deviation values of segment lengths (median ± MAD), the specificity values and the sensitivity values estimated from the audio are reported. In each comparison, where the HQmic was considered as a reference measure, a specificity higher than 0.85 was measured while the sensitivity was lower. We have to stress that the GTal algorithm parameters were chosen to achieve this behavior of sensitivity and specificity values [32]. In fact, the identification of a voiced segment as an unvoiced one, *i.e.*, false negative event, was considered less dangerous with respect to the incorrect labeling of an unvoiced segment as voiced.

Table 2 shows the results of the correlational analyses of the features estimated from high quality samples and smartphone data. Features were estimated using the GTal algorithm. The results obtained with the smartphones placed on the table and held by subjects are very similar. In both subjects, jitter estimated using data from a hand-held smartphone showed a weaker, although significant correlation with features estimated using high quality data. The comparison between the two smartphones, *i.e.*, ${\text{SPmic}}_{\text{hand}}$ and ${\text{SPmic}}_{\text{table}}$, showed high correlation coefficients (see supplementary material).

**Table 1:** GTal, microphones comparison: segment lengths (median ± MAD), specificity (Spec) and sensitivity (Sens) estimated from the different concurrent audio samples. Spec and Sens are defined with respect to voiced segments as identified on the high quality microphone (HQmic).

GTal: Microphones Comparison				
GTal: HQmic *vs.* ${\text{SPmic}}_{\text{hand}}$				
Subj.	${\text{len}}_{\text{HQmic}}$ [ms]	${\text{len}}_{{\text{SPmic}}_{\text{hand}}}$ [ms]	Spec	Sens
1	136 ± 56	160 ± 80	0.89	0.81
2	128 ± 64	168 ± 88	0.85	0.83
GTal: HQmic vs. SPmic${}_{\text{table}}$				
Subj.	${\text{len}}_{\text{HQmic}}$ [ms]	${\text{len}}_{{\text{SPmic}}_{\text{table}}}$ [ms]	Spec	Sens
1	136 ± 56	152 ± 72	0.89	0.77
2	128 ± 64	128 ± 64	0.90	0.81

**Table 2:** GTal, microphones comparison: correlation coefficients between features extracted from the audio acquired with the HQmic and ${\text{SPmic}}_{\text{hand}}$ on overlapping portions of the segments. The corresponding *p*-values are shown in brackets.

GTal: Microphones Comparison			
**Subj.**	**Comparison**	**Feature**	**ρ [*p*-value]**
	HQmic	$meanF_{0}$	1.00 [<$10^{-6}$]
1	*vs.*	$stdF_{0}$	0.96 [<$10^{-6}$]
	${\text{SPmic}}_{\text{hand}}$	Jitter	0.81 [2.3$\times 10^{-6}$]
	HQmic	$meanF_{0}$	0.95 [1.90$\times 10^{-6}$]
1	*vs.*	$stdF_{0}$	0.90 [1.68$\times 10^{-6}$]
	${\text{SPmic}}_{\text{table}}$	Jitter	0.90 [1.69$\times 10^{-6}$]
	HQmic	$meanF_{0}$	1.00 [<$10^{-6}$]
2	*vs.*	$stdF_{0}$	0.92 [<$10^{-6}$]
	${\text{SPmic}}_{\text{hand}}$	Jitter	0.76 [1.72$\times 10^{-6}$]
	HQmic	$meanF_{0}$	1.00 [<$10^{-6}$]
2	*vs.*	$stdF_{0}$	0.99 [<$10^{-6}$]
	${\text{SPmic}}_{\text{table}}$	Jitter	0.91 [<$10^{-6}$]

### 3.2. Evaluation of the Algorithm

The computational time of the algorithm running on the smartphones was approximately equal to 2 min for a speech recording length equal to 30 s.

**Figure 2:**
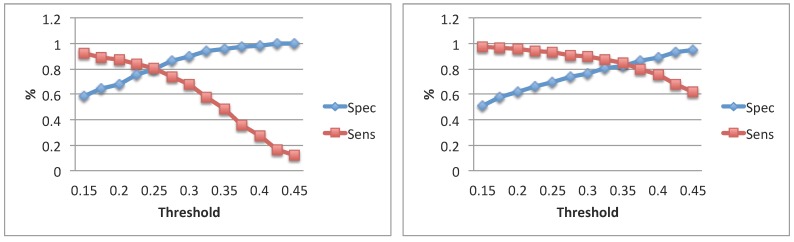
Specificity and sensitivity trends of voiced segmentation in Subject 1 (**left**) and Subject 2 (**right**) regarding the comparison GTal *vs.* ANDal.

The impact of a pitch strength threshold used to detect voiced segments in ANDal was evaluated in terms of obtained specificity and sensitivity values. In Figure 2, the obtained trends are reported. Statistical analysis of the feature distributions revealed that $meanF_{0}$, as estimated by the two algorithms, was not significantly different when varying the pitch strength threshold. This happened in the analysis of Subject 1 data for the threshold values ranging from 0.25 to 0.45 and in the analysis of Subject 2 for all investigated thresholds. Significant differences were found in $stdF_{0}$ and Jitter in both subjects.

The pitch strength thresholds adopted for subsequent analysis (*i.e.*, voiced segments statistics, correlational analysis among features and overall system evaluation) were chosen in order to get a specificity and a sensitivity higher than 0.9 and 0.6, respectively. According to the adopted criteria, two different pitch strength thresholds were chosen for the two subjects: 0.30 for audio related to Subject 1 and 0.45 for audio related to Subject 2.

In Table 3, the parameters describing the differences occurring in the voiced segmentation process between the two algorithms are reported. On average, segment lengths estimated with the ANDal are smaller.

**Table 3:** HQmic, algorithms comparison: segment lengths (median ± MAD), specificity (Spec) and sensitivity (Sens) as estimated on high quality data using both algorithms.

HQmic: Algorithms Comparison				
**HQmic: GTal *vs.* ANDal**				
Subj.	${\text{len}}_{\text{GTal}}$ [ms]	${\text{len}}_{\text{ANDal}}$ [ms]	Spec	Sens
1	136 ± 56	90 ± 60	0.90	0.68
2	128 ± 64	40 ± 30	0.95	0.62

The correlations between features estimated by means of the GTal and those obtained with the ANDal, considering overlapping portions of the segments, are significant. Table 4 reports the results. Higher values are found for $F_{0}$, while lower, although significant, values for features describing $F_{0}$ variability.

**Table 4:** HQmic, algorithms comparison: correlation coefficients regarding the features sets extracted from HQmic by using both ANDal and GTal. The corresponding *p*-values are shown in brackets.

HQmic: Algorithms Comparison			
**Subj.**	**Comparison**	**Feature**	ρ **[*p*-value]**
	GTal	$meanF_{0}$	0.98 [<$10^{-6}$]
1	*vs.*	$stdF_{0}$	0.92 [<$10^{-6}$]
	ANDal	Jitter	0.86 [1.52$\times 10^{-6}$]
	GTal	$meanF_{0}$	0.93 [<$10^{-6}$]
2	*vs.*	$stdF_{0}$	0.81 [<$10^{-6}$]
	ANDal	Jitter	0.71 [3.28$\times 10^{-6}$]

### 3.3. Evaluation of the System

**Figure 3:**
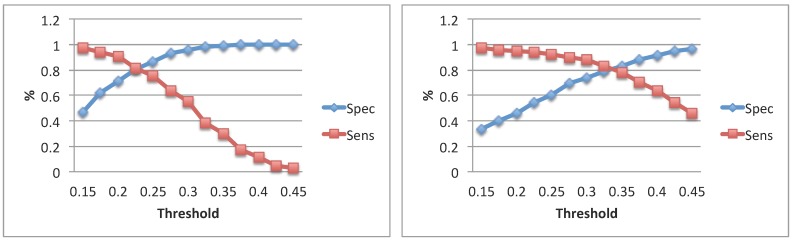
Specificity and sensitivity trends of voiced segmentation in Subject 1 (**left**) and Subject 2 (**right**) regarding the comparison HQmic + GTal *vs.* ${\text{SPmic}}_{\text{hand}}$ + ANDal.

Figure 3 shows specificity and sensitivity curves of the voiced segmentation step as functions of the pitch threshold. The obtained trends are related to the comparison between the high quality system and the smartphone kept in the hand for both subjects. Regarding the $meanF_{0}$ distributions, no statistically-significant differences were found between the two approaches for large intervals of the pitch strength values. Specifically in Subject 1, such a behavior was observed for the threshold ranging from 0.28 to 0.45, while in Subject 2, it was observed for all investigated thresholds ranging from 0.15 to 0.45. Statistically-significant differences were observed for the other features describing $F_{0}$ variability.

For subsequent analysis (*i.e.*, voiced segment statistics and correlational analyses between estimated features), the pitch strength thresholds determined in the algorithm evaluation step with high quality data were used. Table 5 shows the median segment length, the specificity and the sensitivity values estimated by the different systems. On average, segment lengths estimated with the smartphone system are smaller. Specificity values higher than 0.93 were always detected, while sensitivity values were lower. The results were similar for both subjects and both smartphone positions. In Table 6, the correlation coefficients between the features obtained from the different systems and taking into account overlapping portions of the segments are shown. Also in this case, the position of the smartphone did not affect the results. Mean $F_{0}$ was still found to be the most reliable feature, although significant correlations were observed regarding the other features.

**Table 5:** Overall estimation of the system: segment lengths (median ± MAD), specificity (Spec) and sensitivity (Sens) estimated using the two different systems.

System Evaluation				
HQmic + GTal *vs.* ${\text{SPmic}}_{\text{hand}}$ + ANDal				
Subj.	${\text{len}}_{\text{HQmic}}$ [ms]	${\text{len}}_{{\text{SPmic}}_{\text{hand}}}$ [ms]	Spec	Sens
1	136 ± 56	85 ± 35	0.96	0.56
2	128 ± 64	40 ± 25	0.93	0.46
HQmic + GTal *vs.* ${\text{SPmic}}_{\text{table}}$ + ANDal				
subj.	${\text{len}}_{\text{HQmic}}$ [ms]	${\text{len}}_{{\text{SPmic}}_{\text{hand}}}$ [ms]	Spec	Sens
1	136 ± 54	70 ± 30	0.96	0.47
2	128 ± 64	40 ± 20	0.97	0.40

**Table 6:** Overall estimation of the system. Correlation coefficients regarding the investigation about the features extracted from audio coming from the two different systems: HQmic + GTal *vs.* ${\text{SPmic}}_{\text{hand}}$ + ANDal and HQmic + GTal *vs.* ${\text{SPmic}}_{\text{table}}$ + ANDal. The corresponding *p*-values are shown in brackets.

System Evaluation			
**Subj.**	**Comparison**	**Feature**	ρ **[*p*-value]**
	HQmic + GTal	$meanF_{0}$	1.00 [<$10^{-6}$]
1	*vs.*	$stdF_{0}$	0.92 [8.25$\times 10^{-7}$]
	${\text{SPmic}}_{\text{hand}}$ + ANDal	Jitter	0.84 [1.63$\times 10^{-6}$]
	HQmic + GTal	$meanF_{0}$	0.99 [<$10^{-6}$]
1	*vs.*	$stdF_{0}$	0.94 [<$10^{-6}$]
	${\text{SPmic}}_{\text{table}}$ + ANDal	Jitter	0.78 [6.48$\times 10^{-6}$]
	HQmic + GTal	$meanF_{0}$	0.89 [<$10^{-6}$]
2	*vs.*	$stdF_{0}$	0.77 [<$10^{-6}$]
	${\text{SPmic}}_{\text{hand}}$ + ANDal	Jitter	0.79 [<$10^{-6}$]
	HQmic + GTal	$meanF_{0}$	0.91 [<$10^{-6}$]
2	*vs.*	$stdF_{0}$	0.78 [<$10^{-6}$]
	${\text{SPmic}}_{\text{table}}$ + ANDal	Jitter	0.74 [<$10^{-6}$]

### 3.4. Smartphone Model Comparison

The results of the comparison between the two different smartphone models are shown in Table 7. Regarding the feature distributions, no statistically-significant differences were found. Moreover, a high correlation between the extracted features was highlighted.

**Table 7:** Comparison between different smartphone models. Correlation coefficients regarding the investigation about the features extracted from audio coming from the two different smartphone models. The corresponding *p*-values were always smaller than $10^{-6}$.

Comparison between Different Smartphone Models			
**Subj.**	**Algorithm**	**Feature**	ρ
		$meanF_{0}$	1.00
1	ANDal	$stdF_{0}$	0.97
		Jitter	0.90
		$meanF_{0}$	1.00
2	ANDal	$stdF_{0}$	0.82
		Jitter	0.90

### 3.5. Results on Bipolar Data

The patient was two times in a depressive state, four times in a hypomanic state and nine times in euthymic state. This subject never experienced a mixed state. To perform a correlational analysis between mood state and speech feature changes, the observed mood states were ranked in increasing order passing from hypomania to depression.

**Figure 4:**
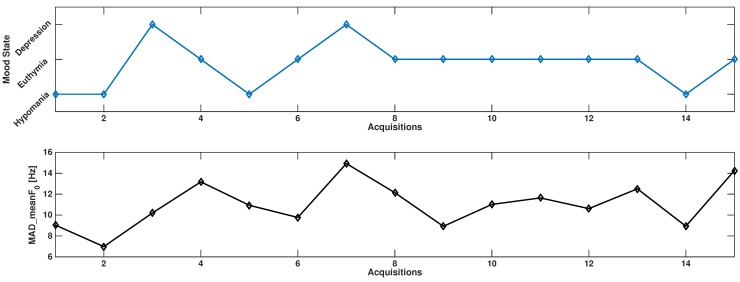
Trend observed in the reported patient: ρ=0.54, *p*-value = 0.0392.

The results indicate a correlation between mood state changes and MAD of the distribution of the $meanF_{0}$, indicated as $MAD\_meanF_{0}$ (ρ=0.54, *p*-value = 0.0392). In Figure 4 the observed trends are shown. No significant correlations were found between the observed features and the QID or the YMRS scales.

## 4. Discussion

In this study, data were processed locally, on a smartphone, instead of a central server. Although a central server solution can provide a more complex analysis of the raw speech signal, the smartphone offers some advantages. Using a smartphone increases user trust because the speech data are stored locally. Moreover, data upload is reduced, occupying 75 kB instead of 6 MB for a 30-s recording. Local processing facilitates the use of the features to provide prompt feedback to the user for the realization of self-management applications without network accessibility. Regarding the processing time, the ever-increasing processing power of mobile phones will decrease the time lag between the end of the task and the availability of the results. In the current release of the application, the estimates of the $F_{0}$ values, the related times, as well as the strengths obtained by the algorithm were sent to a server, for a further processing step. This last processing step detects voiced segments using a threshold approach and estimates the final features along with some statistics related to their distribution across all segments.

The comparative results here presented demonstrate the limits and the possibilities of the proposed system in performing a remote acquisition and analysis of speech samples. The overall quality of the system, in terms of the reliability of the final results, can be obtained by looking at the distributions of the estimated features across all segments. However, we performed several tests to determine the factors that influence the final results. The segmentation results, considering only an audio quality test, demonstrate that good specificity and sensitivity scores can be reached by processing smartphone recordings. Even if both undetected voiced segments and a low percentage of unvoiced segments are present, the quality of audio acquisitions is good enough to perform a robust estimate of features related to $F_{0}$ and its changes. A further analysis can be done to check whether other features, such as vocal tract features and spectral features, can be estimated, as well. Adding noise to the speech signal may reduce the reliability of the results. An analysis taking into account different categories and intensities of noise sources could clarify the robustness of the approach in noisy environments. Several strategies and capabilities of current smartphones could be further explored to reduce the weight of ambient noise, as active noise reduction strategies that exploit a second environmental microphone. The analysis of the performance of the Android algorithm revealed that $meanF_{0}$ can be reliably estimated. On the other hand, the features related to $F_{0}$ variability estimated within each voiced segment by the ANDal show different distributions with respect to those obtained with the GTal. Nonetheless, looking at the correlational analysis using features estimated on overlapping portions of the segments, it is possible to notice that the Android algorithm can reliably estimate those features, as well. These results can be explained by the reduction of segment length that is compatible with the reduction of the sensitivity value. Hence, the reliability of the features describing $F_{0}$ variability, $F_{0}$ standard deviation or jitter seem to be reduced by the detection of shorter segments by the Android algorithm.

It should be stressed that on the mobile application, voiced segments are detected using a threshold applied to the strength parameter estimated by the SWIPE’ algorithm. As a selection criterion, we decided to choose thresholds preferring specificity with respect to sensitivity. In fact, such a choice minimizes the number of analyzed unvoiced segments as opposed to voiced ones. Thresholds cannot be increased too much, since too low a sensitivity could depauperate the informative content of the investigated features. On the other hand, a decrease of the threshold could result in longer segments, but also in an increase of unvoiced segment detection. Therefore, it is important to note that our results show that an improvement of the mobile application could be achieved by modifying the segmentation approach. More specifically, such an improvement could be obtained by using clustering techniques, as described in [27], or by exploiting signal intensity and the zero crossing rate. The study on specificity and sensitivity regarding the ANDal varying the threshold of the pitch strength revealed that this parameter is speaker dependent. Both the Android algorithm and overall system evaluations revealed that no statistically-significant differences were found when analyzing the two $meanF_{0}$ distributions across a large range of pitch strength thresholds. This result indicates that $meanF_{0}$ is robust with respect to errors in the determination of the threshold. This observation led us to hypothesize that this system could be used to monitor this feature in a more general framework, e.g., with different subjects.

Our overall results show that $meanF_{0}$ estimated across all segments is the most reliable feature. The analysis of this feature reflects the long-term changes of $F_{0}$ across all recordings. Short-term variations, with increasing level of detail, are described by $F_{0}$ standard deviation and jitter. Although former features estimation is affected by the voiced segmentation performance of the algorithm, some information can be still obtained. In fact, the correlational analysis revealed that a portion of feature variance can be captured by the system. Further investigations are needed to verify whether the changes of the feature distribution across time, *i.e.*, across different acquisitions, can still be detected.

We reported an example of an application tested on a bipolar subject. Interestingly, a similar trend was found between the measure of variation of average $F_{0}$ as estimated across voiced segments. In this subject, the variability of this prosodic feature, indicating the dynamics of fundamental frequency across all segments, seems to increase passing from hypomania to depression. This behavior was observed elsewhere [17] and may be related to agitated depression. We noticed that the increase of the feature at Week 5 was delayed with respect to a change from euthymia to depression. However, the trends of the proposed scale and of the reported feature were very similar.

Our results are in line with clinical models of bipolar disorders that consider depressive and maniac symptoms at the extremities of a continuous scale, while euthymia is in the middle [34]. This is not only true for pure mood symptoms, but also for the sexuality and instinct dimensions, for the psychomotor activity dimension and for the energy level dimension (from extremely high in mania, to the extremely low in depression). For other types of symptoms, this dimensional trend is not so clear (e.g., sleep, appetite). The mixed state condition does not fit the dimensional model, since it includes both the symptoms of mania or hypomania and depression at the same time. However the idea of having depression, euthymia, hypomania and mania on a single (or multiple) dimension(s) is a quite effective descriptive tool.

On this subject, no correlations were found among the estimated features and QID or YMRS scales. Further investigations may clarify whether a dose-response relationship could be highlighted and if it is specifically related to a given subtype of symptoms. To test this hypothesis, a higher number of subjects and a higher number of acquisitions for each subject are required. We have to underline that the ordinal scale proposed, linking mania with depression, takes into account the information of both scales and merges the variability observed in both clinical scales. This could be a possible reason to explain why the former scale resulted in a significant correlation. Finally, we do not have data on the mixed state. Given the fact that this peculiar state is not easily described on a linear dimension model, it is difficult to formulate a hypothesis on the behavior of $F_{0}$ in such a condition. However, we believe that given the heterogeneity of the mixed state, also the $F_{0}$ behavior will be variable. In particular, if we accept the hypothesis that the $F_{0}$ is influenced by a specific domain of symptoms (e.g., pure mood) and not by the symptomatology as a whole, we may infer that in the mixed state, $F_{0}$ will behave as depression (or mania) if the given domain is in the depressive (or maniac) extreme of the dimension.

## 5. Conclusions

This work shows that the quality of audio acquisitions from smartphone devices can be used to estimate different features describing the speech $F_{0}$. An Android application was developed to estimate $F_{0}$ and its changes across time locally on a smartphone. The mean value of $F_{0}$ estimated for each voiced segment can be reliably obtained. The tests performed indicated that an improvement of the voiced segmentation procedure could allow one to estimate speech features related to $F_{0}$ variability within each voiced segment. A case study on a bipolar patient shows the potentiality of this approach.

## Supplementary Files

Supplementary File 1
