# Supplementary material for: Smartphone Application for the Analysis of Prosodic Features in Running Speech with a Focus on Bipolar Disorders: System Performance Evaluation and Case Study
Source: Sensors (Basel). 2015 Nov 6;15(11):28070–87. doi: 10.3390/s151128070 (PMC4701269; doi:10.3390/s151128070)
Supplement: Supplementary File 1 [file sensors-15-28070-s001.pdf]

OPEN ACCESS

sensors

ISSN 1424-8220

www.mdpi.com/journal/sensors

Supplementary Information

Smartphone Application for the Analysis of Prosodic Features in Running Speech with a Focus on Bipolar Disorders: System Performance Evaluation and Case Study. *Sensors* 2015, *15*, 28070-28087

Andrea Guidi [1,2,†,*], Sergio Salvi [3,†], Manuel Ottaviano [3], Claudio Gentili [4,5], Gilles Bertschy [6], Danilo de Rossi [1,2], Enzo Pasquale Scilingo [1,2] and Nicola Vanello [1,2,†]

1. Dipartimento di Ingegneria dell'Informazione, University of Pisa, Via G. Caruso 16, Pisa 56122, Italy; E-Mails: d.derossi@centropiaggio.unipi.it (D.R.); e.scilingo@centropiaggio.unipi.it (E.P.S.); nicola.vanello@unipi.it (N.V.)
2. Research Center "E. Piaggio", University of Pisa, Largo L. Lazzarino 1, Pisa 56122, Italy
3. Life Supporting Technologies, Universidad Politécnica de Madrid , Avd. Complutense 30, Madrid 28040, Spain; E-Mails: ssalvi@lst.tfo.upm.es (S.S.); mottaviano@lst.tfo.upm.es (M.O.)
4. Department of Surgical, Medical, Molecular Pathology and Critical Care, University of Pisa, Via Savi 10, Pisa 56126, Italy; E-Mail: c.gentili@unipd.it
5. Department of General Psychology, University of Padua, Via Venezia 8, Padua 35131, Italy
6. Department of Psychiatry and Mental Health, Strasbourg University Hospital, INSERM U1114, Translational Medicine Federation, University of Strasbourg, Strasbourg 67000, France; E-Mail: gilles.bertschy@chru-strasbourg.fr

† These authors contributed equally to this work.

* Author to whom correspondence should be addressed; E-Mail: andrea.guidi@for.unipi.it; Tel.: +39-50-2217462; Fax: +39-50-2217522.

Table S1. Correlation coefficients between features extracted from the audio acquired with the $SPmic_{hand}$ and $SPmic_{table}$ on overlapping portions of the segments. The corresponding *p*-values are shown in brackets.

GTal: Microphones Comparison			
**Subj.**	**Comparison**	**Feature**	**ρ [p-value]**
1	$SPmic_{hand}$	$meanF_0$	1.00 [$<10^{-6}$]
	vs.	$stdF_0$	0.99 [$<10^{-6}$]
	$SPmic_{table}$	$Jitter$	0.92 [$<10^{-6}$]
2	$SPmic_{hand}$	$meanF_0$	1.00 [$<10^{-6}$]
	vs.	$stdF_0$	1.00 [$<10^{-6}$]
	$SPmic_{table}$	$Jitter$	0.98 [$<10^{-6}$]

© 2015 by the authors; licensee MDPI, Basel, Switzerland. This article is an open access article distributed under the terms and conditions of the Creative Commons Attribution license (http://creativecommons.org/licenses/by/4.0/).
